# Exploring the Barriers in the Uptake of the Dutch MRSA ‘Search and Destroy’ Policy Using the Cascade of Care Approach

**DOI:** 10.3390/antibiotics11091216

**Published:** 2022-09-08

**Authors:** Annette C. Westgeest, Emile F. Schippers, Martijn Sijbom, Leo G. Visser, Mark G. J. de Boer, Mattijs E. Numans, Merel M. C. Lambregts, on behalf of the MRSA Network Holland West

**Affiliations:** 1 Department of Infectious Diseases, Leiden University Medical Center, 2333 Leiden, The Netherlands; 2 Department of Internal Medicine, Haga Teaching Hospital, 2545 The Hague, The Netherlands; 3 Department of Public Health and Primary Care, Leiden University Medical Center, 2333 Leiden, The Netherlands; 4 Department of Clinical Epidemiology, Leiden University Medical Center, 2333 Leiden, The Netherlands

**Keywords:** methicillin-resistant *Staphylococcus aureus*, MRSA, decolonization, search and destroy, cascade of care, CA-MRSA

## Abstract

The Dutch ‘search and destroy’ policy consists of screening patients with an increased risk of methicillin-resistant *Staphylococcus aureus* (MRSA) carriership and subsequent decolonization treatment when carriership is found. Decolonization therapy of individual MRSA carriers is effective. However, the effectiveness of the national ‘search and destroy’ policy is dependent on the entire cascade of care, including identification, referral, and subsequent treatment initiation in MRSA carriers. The aim of this study was to evaluate the leakages in the cascade of MRSA decolonization care. We assessed familiarity with the ‘search and destroy’ policy and the barriers in the uptake of MRSA eradication care using a questionnaire among 114 Dutch general practitioners. The main reasons for treatment were planned hospital visits, occupational reasons, and infections. The main reasons for refraining from eradication treatment were unfamiliarity with the ‘search and destroy’ policy and the assumption that MRSA carriership is often self-limiting. To optimize the continuity of the cascade of care, interventions should be aimed at supporting general practitioners and facilitating treatment and referral.

## 1. Introduction

Antimicrobial resistance is a global health threat that causes millions of deaths [1]. The WHO has declared that antimicrobial resistance is one of the top ten global public health threats facing humanity [2]. Methicillin-resistant *Staphylococcus aureus* (MRSA) is a major actor in the field of antimicrobial resistance. In 2019, 100.000 deaths and 3.5 million disability-adjusted life-years (DALYs) were attributable to infections with MRSA [3]. Colonization with MRSA leads to increased infection rates of up to 25% [4,5,6]. 

Colonization and infection rates are known to vary throughout the world. Historically, in the Netherlands, MRSA infection rates are low. Less than 5% of invasive *Staphylococcus aureus* isolates are resistant to methicillin. Together with the Nordic European countries, the Dutch prevalence of MRSA is the lowest in the world [7]. The estimated nasal colonization rate in the Dutch population is 0.03–0.17%, compared to 0.9–1.5% in the US [8]. 

The healthcare system in the Netherlands has executed a national ‘search and destroy’ policy since 1988, which is outlined in the guidelines of the Dutch Working Party on Infection Prevention (WIP) [9]. The policy consists of the screening and preemptive isolation of patients with an increased risk of MRSA carriership when hospitalized and subsequent decolonization treatment when persistent carriership is found [10,11,12]. Examples of an increased risk are preceding events such as hospitalization in a country where MRSA is endemic, or a confirmed MRSA-carrying household contact. The aim of the policy, which is endorsed by the Dutch health council, is to keep the MRSA prevalence and the associated disease burden low [13]. Cost-effectiveness was confirmed in the years thereafter, with an estimated saving of up to EUR 400 per hospital per year [10,14]. 

As part of this ‘search and destroy’ policy, decolonization treatment in MRSA carriers has proven to be an effective preventive strategy in reducing infection and hospitalization rates [15]. The success rate of decolonization treatment, defined as three consecutive negative MRSA swabs from nose, throat, and perineum, is as high as 86% [16]. However, the effectiveness of the policy is also dependent on the initial identification of carriership and the initiation of treatment. 

**Figure 1:**
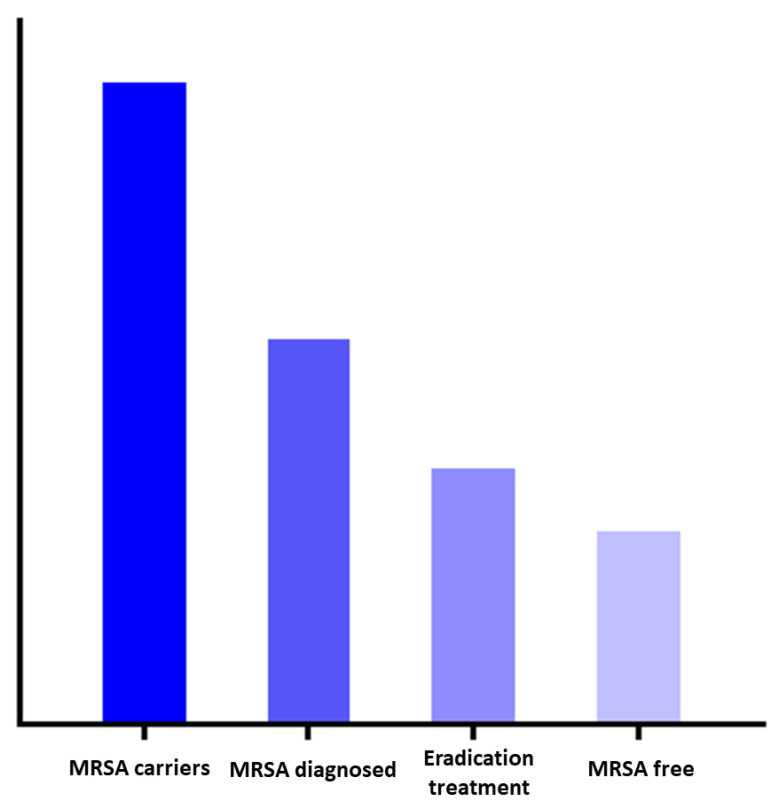
Conceptual graphic of the cascade of care in MRSA decolonization. Legend: The first column addresses the total number of MRSA carriers in the Netherlands. The second column represents the proportion of carriers that is diagnosed. The third column addresses the MRSA carriers that are diagnosed and undergo eradication treatment. The last column represents the success rate of complicated MRSA eradication treatment. In every step of this conceptual cascade of care, there is the potential for leakage. As this figure represents a conceptual model, the columns are not quantified.

Therefore, the effectiveness of the national policy relies on the correct execution of several consecutive steps in a so-called cascade of care and involves several healthcare professionals. In HIV care, a similar approach was taken and led to the clarification of the culprits in the uptake of combination anti-retroviral therapy (cART) [17]. Following this example, this approach was applied to tuberculosis and hepatitis C [18,19]. We hypothesize that the same approach is applicable to MRSA decolonization care as well (Figure 1). Within the MRSA decolonization cascade of care, individuals may be lost, which is referred to as leakage, and is analogous to the cART roll-out strategies. Understanding at which steps this leakage occurs will provide information to optimize MRSA eradication strategies [20]. 

The aim of our current study was to evaluate the leakages within the cascade of MRSA decolonization care and the main reasons for them. We carried out a questionnaire study amongst general practitioners (GPs) to gain insight into their familiarity with the ‘search and destroy’ policy and to evaluate barriers in the uptake of MRSA eradication care. The knowledge generated will help to determine specific targets that can be addressed to keep MRSA prevalence low and to contribute to a reduced burden of antimicrobial resistance.

## 2. Methods

The questionnaire study was executed in primary care as GPs hold a central position in the Dutch healthcare system. All Dutch citizens are registered with a general practitioner (GP), who is the first point of contact in case of illness and acts as a gatekeeper to secondary care. With regard to MRSA carriership, the GPs are often the first healthcare professionals to be in contact with patients at risk or to detect MRSA carriership. 

### 2.1. Questionnaire Development and Distribution

The regional MRSA Network developed a questionnaire that was reviewed by a panel consisting of a general practice specialist and an infectious disease specialist (Supplementary File S1). The questionnaire included 14 questions on the ‘search and destroy’ policy, the screening of risk patients, the difference between complicated and uncomplicated carriership, and eradication therapy. Two case vignettes were included to assess daily practice (Box 1). The target population consisted of GPs in the Netherlands. The questionnaire was hosted on Formdesk, a web-based survey platform, and was distributed via different networks of GPs and newsletters from participating hospitals. The majority of the recipients were situated in the western part of the Netherlands. There was the possibility of responding anonymously. The questionnaire was accessible between 7 March 2022 and 13 June 2022. Descriptive statistics were used to summarize the data derived from the Formdesk software.

Box 1 **Case vignettes.**

Case A:
A 26 years-old healthy male was admitted in the hospital during a holiday in Spain because of a trauma. After returning in the Netherlands, you perform culture swabs from nose, throat and perineum. The nasal culture is positive for MRSA. There are no skin lesions. There are no hospital visits planned.
Case B:
A 56 years-old male with a history of heart failure and chronic kidney disease, was screened for MRSA carriership by you following a hospital admission. He is MRSA positive in nose, throat and perineum.

Legend: Two clinical case vignettes were included in the questionnaire. Case A describes a patient with uncomplicated carriership. Case B describes a patient with complicated carriership. The guideline recommends treatment with topical therapy in case A and treatment with additional (systemic) antibiotics in case B.

### 2.2. Definitions

The Dutch national guideline on the treatment of MRSA carriers recommends different eradication treatments depending on the type of carriership. Uncomplicated MRSA carriership is defined as having all of the following features: (i) the presence of MRSA exclusively located in the nose, (ii) no active infection with MRSA, (iii) in vitro sensitivity for mupirocin, (iv) the absence of active skin lesions, (v) the absence of foreign material that connects an internal body site with the outside (e.g., urine catheter or external fixation material), and (vi) no previous failure of decolonization treatment. All other cases are considered to be complicated colonization [21]. Uncomplicated carriership is treated with topical therapy (mupirocin topically applied to the nares and disinfecting shampoo) and hygienic measures. In the case of complicated MRSA carriage, additional systemic antimicrobial therapy with a combination of two antibiotic agents is recommended. Furthermore, the guideline recommends the screening of household contacts (and sometimes pets) and the simultaneous treatment of colonized household contacts [21].

## 3. Results

The questionnaire was completed by 114 Dutch GPs. The majority of the GPs (98/114, 86%) performed screening for MRSA carriership. Recent admission to a hospital abroad was more often considered to be the reason for screening in older patients with comorbidity (89/114, 78%) compared to younger patients without comorbidity (77/114, 68%). A previous infection with MRSA was considered to be a reason for screening by 55/114 (48%) of the GPs and a positive household contact by 39/114 (34%) of the GPs.

The majority of the respondents, 98/114 (86%), reported having 1- 3 new MRSA cases per year. Fifteen GPs (15/114, 13%) stated that they had never had a single patient in his/her practice. The median prevalence of MRSA carriers per practice was 2 (interquartile range 0–4).

With regard to the familiarity with the explicit ‘search and destroy’ policy in the Netherlands, 98/114 (86%) of the GPs indicated that they were not familiar with this policy.

### 3.1. Initiation of Eradication Therapy and/or Referral for Treatment

Almost half of the GPs (52/114, 46%) estimated that <20% of the MRSA carriers in their practice received eradication therapy. With respect to the indication for eradication treatment, most of the GPs (58/114, 51%) stated that only specific MRSA carriers should be eligible for eradication treatment, namely if there is a specific reason (e.g., frequent hospital visits) (58/58, 100%), if the patient is a healthcare worker with clinical duties (52/58, 90%), if the patient has an infection with MSRA (42/58, 72%), or if the patient insists on treatment (10/58, 17%).

The most important reasons to refrain from eradication therapy were: the potentially self-limiting nature of MRSA carriership (59%), unfamiliarity with the Dutch ‘search and destroy’ policy (25%), the burden of treatment for the patient (23%), the lack of any recommendation being known GP protocols (18%) and the patients’ explicit request not to be treated (18%) (Table 1).

### 3.2. Treatment of MRSA Carriership

Forty-four respondents (44/114, 39%) had treated patients with (complicated or uncomplicated) MRSA carriership themselves—in all cases or in selected cases. When treating a patient for MRSA carriership, 10/44 (23%) of the responding GPs included the screening and treatment of household contacts in the initial treatment attempt, 5/44 (11%) included the household contacts only after a failed treatment attempt, and 12/44 (27%) never included household contacts. Other GPs (17/44, 39%) stated that they asked an expert for advice. The most important reasons to refrain from referring an MRSA carrier to the hospital were unfamiliarity with the existence of MRSA outpatient clinics (55/114, 48%), feeling competent in the self-performance of treatment (19/114, 17%), and the absence of this recommendation in the guideline (17/114, 15%) (Table 2).

Two cases were presented in the questionnaire: case A was the description of a young patient with an uncomplicated carriership, and case B was a case of a complicated carriership (Box 1). Of the respondents, 40/114 (35%) were aware of the difference between ‘complicated’ versus ‘uncomplicated’ MRSA colonization. Respectively, 37 (33%) and 3 (3%) of the GPs would refrain from treatment in case A and B, 15 (13%) and 56 (49%) would refer the patient to a hospital for treatment, and 29 (25%) and 31 (27%) would first consult a specialist. Of the GPs that would initiate treatment in these cases themselves (17 in case A and 14 in case B), the treatment prescription was in accordance with the treatment guideline for 12/17 (71%) in case A (uncomplicated carriership) and for 8/14 (57%) in case B (complicated carriership). In both cases, four GPs (24%, 29%) indicated to add or refrain from systemic antibiotics where this was not in accordance with the guideline (Supplementary File S2).

## 4. Discussion

The main finding of this study is that there is significant leakage in the cascade of MRSA decolonization care. Firstly, the vast majority of the responding GPs are not familiar with the explicit ‘search and destroy’ policy. Secondly, when evaluating a patient with MRSA carriage, many assumptions are made to refrain from eradication treatment. Thirdly, eradication treatment is not always in accordance with the guideline. The conceptual steps of the cascade of MRSA colonization care are visualized in Figure 1.

For optimal effect of the strategy, adherence to each consecutive step is crucial. Based on our findings, the uptake of decolonization care in the Netherlands, as part of the ‘search and destroy’ policy, is not flawless. All subsequent process steps in the cascade have the potential for improvement. We summarized the main leakages of the cascade and the possible solutions in Table 3. The most apparent opportunity for the improvement of its implementation is through expanding familiarity with the ‘search and destroy’ policy. All three steps in the cascade could benefit from the training/education of both the patients and the professionals. In addition, incorporating the policy in the GP practice guidelines should be considered in order to support the entire process from screening to successful eradication. The current national MRSA decolonization guideline is primarily targeted at medical specialists, and the recommendations for screening and treatment have not yet been translated to the Dutch GP guidelines [22]. At the patient level, financial barriers exist that could be targeted by waving the excess fee for MRSA decolonization care.

Despite the described leakages in the identification and treatment of MRSA carriership, the MRSA prevalence is low in our country compared to surrounding countries. The estimated nasal colonization rate in the Netherlands was 0.03–0.17% in 2010–2017 [23]. It is generally accepted that this is largely attributed to the ‘search and destroy’ policy [11,24,25,26,27]. The policy seems to be effective, despite the leakages we found in the decolonization cascade. The effectivity of the policy as a whole is only partly determined by the uptake of screening and decolonization therapy. Another important arm of the ‘search and destroy’ policy—the preemptive isolation of patients at risk—was not assessed in the current study.

There has been debate about the rigorous ‘search and destroy’ policy in the past. Up to the present day, it is the subject of discussion whether healthy carriers that do not have any connections with hospital healthcare should be treated [21]. This is reflected in our results, where the GPs were less inclined to treat a young healthy MRSA carrier compared to an older patient with comorbidity. Although this is a leak in the cascade of care, not treating this subset of MRSA carriers is justifiable as stated in the Dutch guideline. Overall, the last report of the Dutch health council to the Ministry of Health in 2006, advising the continuation of the ‘search and destroy’ policy, is still valid [13]. Efficacy and cost-effectiveness have been demonstrated in the past [10,14]. The semi recent history of the United Kingdom is an extra confirmation of the effectiveness of this approach. In the UK, a similar strict MRSA policy was carried out in the 1980s. After the policy was tempered in the 1990s, the percentage of methicillin resistance in *Staphylococcus aureus* bacteremia increased steeply from <2% to >30% [28,29]. This percentage is now lower due to rigorous measures on hygiene and the mandatory reporting of MRSA, as part of a major public health infection prevention campaign [30].

To our knowledge, this study is the first to map the MRSA cascade of care. Although the methodology does not enable the quantification of the leakage within the different cascade steps, it does provide specific targets for the optimization of the cascade. The central position of GPs in the healthcare system is a characteristic of the Netherlands. However, the targets for optimization and proposed interventions could be translated to settings where GPs do not hold a central position, with a greater focus on hospitals.

A limitation of the study is the fact that all results were self-reported. Answers are subject to bias, and potential targets may have been missed. Furthermore, the majority of the respondents were from one region in the Netherlands, which is mainly an urbanized area. In regions with more agriculture and more livestock-associated MRSA, knowledge about MRSA and attitudes towards MRSA carriership may differ [31]. Another limitation is the fact that the response rate was unknown as a result of the various ways (e.g., newsletters) that the questionnaire was distributed. Assuming that the GPs with an affinity with MRSA were more inclined to respond, bias would be in favor of an overall knowledge of the policy. We believe that the identified barriers are valid, even if the response rate were to be relatively low.

## 5. Conclusions

In conclusion, the results of this survey and the derived cascade of care reveal that there are barriers in the uptake of the ‘search and destroy’ MRSA policy in the Netherlands. Low health-provider familiarity with the policy, lack of GP guidelines on the topic, and financial constraints are key factors. To optimize the continuity of the cascade of care, interventions should be aimed at supporting healthcare professionals in the execution of the ‘search and destroy’ policy. Eventually, this will be beneficial both on the population level and for the individual patient.

## Figures and Tables

**Table 1:** The attitude of GPs towards indication for treatment of MRSA carriership.

	Frequency n/n (%)
**Indication for eradication treatment**	
In all MRSA carriers	18/114 (16)
In selected cases	58/114 (51)
*Planned/expected hospital visits*	*58/58 (100)*
*Infections with MRSA*	*42/58 (72)*
*Occupational reason (e.g., healthcare worker)*	*52/58 (90)*
*Patients’ request*	*10/58 (17)*
In none of the MRSA carriers	1/114 (1)
Unknown	37/114 (32)
Reasons to refrain from treatment *	
Potential self-limiting nature of MRSA carriership	57/96 ** (59)
Unfamiliarity with the policy	24/96 (25)
Treatment burden for patients	22/96 (23)
Lack of recommendation in the GP guideline	17/96 (18)
Patients’ request	17/96 (18)
Absence of benefit for the patient	11/96 (11)
Sense of incompetence to guide a treatment	10/96 (10)
Absence of benefit for the society	5/96 (5)
Costs for the patient	4/96 (4)
Other ***	19/96 (20)
Other ***	19/96 (20)

Legend: Indications for MRSA eradication according to Dutch general practitioners and reasons not to initiate treatment or refer for treatment. * Multiple answers possible. ** Eighteen GPs who answered in the previous question that all MRSA carriers have an indication for eradication treatment were not asked for reasons to refrain from treatment. *** Other reasons mentioned in free text: not a task for the GP, assumption of no curation, never considered, patient in palliative setting. GP = general practitioner.

**Table 2:** Treatment of MRSA carriers.

	Frequency n/n (%)
**Estimated proportion of carriers in a GP practice** that receive treatment *	
<20%	52/114 (46)
20–40%	8/114 (7)
40–60%	11/114 (10)
60–80%	12/114 (11)
80–100%	25/114 (22)
Unknown	6/114 (5)
**Treatment by GP or referral to hospital**	
Treatment by GP in all cases	12/114 (11)
Referral to a hospital in all cases	40/114 (35)
Treatment by GP in selected cases	32/114 (28)
*Uncomplicated carriership*	*23/32 (72)*
*Patient preference for GP treatment*	*9/32 (28)*
*Other*	*8/32 (25)*
None of the above	27/114 (24)
Reasons not to refer to a hospital **	
Unfamiliar with the existence of MRSA outpatient clinics	55/114 (48)
Competent in self-performance	19/114 (17)
Lack of recommendation in GP protocol	17/114 (15)
Patients’ request not to be referred	13/114 (11)
Costs for the patient ***	13/114 (11)
Administrative burden of a referral	3/114 (3)
Other ****	33/114 (29)
Unknown	10/114 (9)

Legend: * Estimation of the proportion of known MRSA carriers in the practice that are receiving eradication therapy or have received eradication treatment in the past. ** Multiple answers possible. *** In the Netherlands, the health insurance charges the patient an obligatory deductible excess for hospital care. **** Other reasons mentioned in free text were: consultation of specialist is sufficient, never considered, palliative settings, refusal of hospital, or not specified. GP = general practitioner.

**Table 3:** Leakages in cascade of MRSA decolonization care and possible solutions.

Cascade Leakage	Causes	Potential Interventions	
**From colonization to diagnosis**	Unfamiliarity with the ‘search and destroy’ policy		Education of patients and the public Education of healthcare professionals Accessible information on MRSA policy
	Unfamiliarity with screening indications		Education of healthcare professionals Incorporate screening advice in culture results that are provided by microbiology lab (in case of MRSA infection)
	Financial burden associated with screening (excess fee)		Exempt screening costs from the patients’ obligatory deductible excess fee
**From diagnosis to initiation of treatment**	Unfamiliarity of GPs with the existence of MRSA outpatient clinics		Promote MRSA outpatient clinics through newsletters and by incorporating referral details in culture results
	Lack of indications for decolonization treatment in the GP protocol		Include a paragraph on eradication treatment in the GP skin/soft tissue infection guideline
	Perceived incompetence to start treatment by GP		Facilitate easy consultation with microbiologist or infectiologist Incorporate treatment protocol in the GP guideline
	Patients’ request to refrain from treatment		Patient education (e.g., website and/or patient information folders)
	Financial burden associated with treatment/referral (excess fee)		Exempt treatment/referral costs from the obligatory deductible excess fee
**From treatment to** **successful eradication**	Knowledge deficit, e.g., different treatment of complicated and uncomplicated carriership		Education of healthcare professionals Facilitate easy consultation with an infectiologist or microbiologist Provide patient instruction materials
	Lack of treatment protocol in GP guidelines		Incorporate treatment protocol in GP guidelines

Legend: Causes of leakages in the cascade of MRSA decolonization care derived from the questionnaire and possible solutions devised by the MRSA Network. GP = general practitioner.

## Data Availability

Not applicable.

## Supplementary Materials

The following are available online at https://www.mdpi.com/article/10.3390/antibiotics11091216/s1, File S1. Questionnaire. File S2. GP’s responses to clinical cases
