# Supplementary material for: Exploring the Barriers in the Uptake of the Dutch MRSA ‘Search and Destroy’ Policy Using the Cascade of Care Approach
Source: Antibiotics (Basel). 2022 Sep 8;11(9):1216. doi: 10.3390/antibiotics11091216 (PMC9495217; doi:10.3390/antibiotics11091216)
Supplement: Supplementary file 1 [file antibiotics-1904614-supplementary.pdf › antibiotics-1904614-supplementary.pdf]

Supplementary File S1. Questionnaire

The original questionnaire was in Dutch. For publication purposes, it was translated to English.

1. Do you ever have patients with a positive MRSA culture?
   - ☐ Never
   - ☐ Less than once a year
   - ☐ 1-3 times a year
   - ☐ More than 3 times a year

2. How many patients from your practice are proven MRSA carrier at this moment? (estimation)
   ……………………… patients

3. What proportion of the MRSA carriers from your practice are treated for MRSA carriership now or in the past? (estimation)
   - ☐ 80-100%
   - ☐ 60-80%
   - ☐ 40-60%
   - ☐ 20-40%
   - ☐ < 20%

4. Are you familiar with the difference between complicated and uncomplicated MRSA carriership?
   - ☐ Yes
   - ☐ No

5. Do you ever screen patients for MRSA carriership?
   - ☐ No, never
   - ☐ Yes, if: ……………………………………………………………………………………………………

6. Which of the following patients would you screen for MRSA carriership? (multiple answers possible)
   - ☐ A 27 years-old healthy male who was hospitalized for 3 days in Spain because of a trauma
   - ☐ A 20 years-old student who has a MRSA positive household contact
   - ☐ A 60 years-old male who was admitted at the ICU in Spain because of a myocardial infarction
   - ☐ A 40 years-old female who had a MRSA cultured from a furuncle one month ago
   - ☐ None of the above

7. In your opinion, when does a patient who is MRSA carrier qualify for eradication therapy?
   - ☐ Always
   - ☐ Sometimes, namely in case of: (multiple answers possible)
     - o A specific reason, e.g. when frequent hospital visits are expected to come
     - o The patient is suffering from MRSA infections
     - o The patient is a health care worker with clinical duties
     - o The patients insists
     - o Other: …………………………………………………………………………………….
   - ☐ Never
   - ☐ I do not know

8. If not always, what are reasons for you to refrain from MRSA eradication treatment? (multiple answers possible)
   - ☐ It is not in the GP guideline
   - ☐ I was not familiar with the recommendation of eradication of MRSA carriership
   - ☐ In my opinion it is not useful for the patient
   - ☐ In my opinion it is not useful for the society
   - ☐ The costs (own risk) for the patient
   - ☐ Patients do not wish to be treated
   - ☐ MRSA carriership can resolve on its own
   - ☐ The eradication treatment is too much of a burden for the patient
   - ☐ I do not feel competent to guide a MRSA eradication treatment
   - ☐ Other: ……………………………………………………………………………………………………….

The following questions are about treating yourself or referring to a MRSA outpatient clinic. These are outpatient clinics where MRSA patients are treated by an infectiologist/microbiologist.

9. Do you perform eradication therapy of MRSA carriers yourself?
   - ☐ I always treat myself, I never refer to a hospital.
   - ☐ I always refer to a hospital, I never perform MRSA eradication myself.
   - ☐ I treat some patients myself and refer other patients to the hospital.
   - ☐ I never perform this treatment myself and never refer to a hospital either.

10. If option 3 at question 9: which patients do you treat yourself?
    - o Patients with uncomplicated carriership
    - o Patients who do not want to be referred to an outpatient clinic
    - o Patients who still need to pay their own risk (health insurance)
    - o Other: ……………………………………………………………………………………………

11. What are reasons for you to refrain from referral of patients with MRSA carriership? (Multiple answers possible)
    - ☐ I did not know of the existence of MRSA outpatient clinics
    - ☐ It is not recommended in the GP protocol to refer patients for an eradication treatment
    - ☐ I feel competent in performing the treatment myself
    - ☐ Patients do not wish to be referred
    - ☐ The costs for the patient
    - ☐ The administrative burden that comes with a referral
    - ☐ Other: ………………………………………………………………………………………………………

12. Are you familiar with the ‘search and destroy’ policy with regards to MRSA? This ‘search and destroy’ policy means patients with high risk of MRSA colonization need to be screened and that we aim at eradication treatment of MRSA carriers.
    - ☐ Yes
    - ☐ No

Now we want to present two cases.

13. Case A: A 26 years-old healthy male was admitted in the hospital during a holiday in Spain because of a trauma. After returning in the Netherlands, you perform culture swabs from nose, throat and perineum. The nasal culture is positive for MRSA. There are no skin lesions. There are no hospital visits planned.
    What do you recommend with regards to the MRSA carriership?
    - ☐ No eradication treatment
    - ☐ I treat the MRSA carriership myself
    - ☐ I refer the patient to the outpatient clinic
    - ☐ Other: ……………………………………………………………………………………………………..

    In case of treatment yourself, which treatment do you prescribe?

    - ☐ Mupirocin nose cream + disinfecting soap + hygienic measures
    - ☐ The above in combination with systemic antibiotics
    - ☐ Other: ……………………………………………………………………………………………

14. Case B: A 56 years-old male with a history of heart failure and chronic kidney disease, was screened for MRSA carriership by you following a hospital admission. He is MRSA positive in nose, throat and perineum.
    What do you recommend with regards to the MRSA carriership?
    - ☐ No eradication treatment
    - ☐ I treat the MRSA carriership myself
    - ☐ I refer the patient to the outpatient clinic
    - ☐ Other: ……………………………………………………………………………………………………..

    In case of treatment yourself, which treatment do you prescribe?

    - ☐ Mupirocin nose cream + disinfecting soap + hygienic measures
    - ☐ The above in combination with systemic antibiotics
    - ☐ Other: ……………………………………………………………………………………………

Supplementary File S2. GP's responses to clinical cases

Case	Frequency n/n (%)
**Case A. Treatment of uncomplicated carriership**	
No eradication treatment	37/114 (33)
Treatment by GP	17/114 (15)
Topical therapy + hygienic measures	*12/17 (71)*
Topical therapy + hygienic measures + systemic therapy	*4/17 (24)*
Other	*1/17 (6)*
Referral to hospital/MRSA clinic	15/114 (13)
Consultation with specialist	29/114 (25)
Other	15/114 (13)
**Case B. Treatment of complicated carriership**	
No eradication treatment	3/114 (3)
Treatment by GP	14/114 (12)
Topical therapy + hygienic measures	*4/14 (29)*
Topical therapy + hygienic measures + systemic therapy	*8/14 (57)*
Other	*2/14 (14)*
Referral to hospital/MRSA clinic	56/114 (49)
Consultation with specialist	31/114 (27)
Other	10/114 (9)

Legend. Case A. *A 26 years-old healthy male was admitted in the hospital during a holiday in Spain because of a trauma. After returning in the Netherlands, you perform culture swabs from nose, throat and perineum. The nasal culture is positive for MRSA. There are no skin lesions. There are no hospital visits planned. Case B. A 56 years-old male with a history of heart failure and chronic kidney disease, was screened for MRSA carriership by you following a hospital admission. He is MRSA positive in nose, throat and perineum.*
